# Advancement of electric vehicle technologies, classification of charging methodologies, and optimization strategies for sustainable development - A comprehensive review

**DOI:** 10.1016/j.heliyon.2024.e39299

**Published:** 2024-10-12

**Authors:** Vikram Goud Madaram, Pabitra Kumar Biswas, Chiranjit Sain, Sudhakar Babu Thanikanti, Praveen Kumar Balachandran

**Affiliations:** a Department of Electrical Engineering, National Institute of Technology Mizoram, Aizawl, 796012, India; b Electrical Engineering Department, Ghani Khan Choudhury Institute of Engineering & Technology, Malda, West Bengal, India; c Department of Electrical and Electronics Engineering, Chaitanya Bharati Institute of Technology, Hyderabad, 500075, India; d Centre for Cyber-Physical Food, Energy and Water Systems, University of Johannesburg, Johannesburg 2006, South Africa; e Department of Electrical, Electronic and Systems Engineering, Faculty of Engineering and Built Environment, Universiti Kebangsaan Malaysia, Selangor, Malaysia; f Department of Electrical and Electronics Engineering, Vardhaman College of Engineering, Hyderabad, TG, 501218, India

**Keywords:** Electric vehicle, Charging technologies, Converter technologies, Hybrid electric vehicles

## Abstract

This comprehensive review covers the latest EV technologies, charging methods, and optimization strategies. Electric and hybrid vehicles are compared, explaining their operation and effects on energy, efficiency, and the environment. The review covers new EV charging technologies. Conductive charging (CC), the most popular method due to its simplicity and cost, is tested. Wireless power transfer (WPT) systems, which charge without cables, and their integration into urban infrastructure are also examined. Battery swap stations (BSS), which quickly recharges depleted batteries, are also reviewed for their viability and adoption issues. Electric vehicle standards like charging rate and system configuration are covered in this paper. These standards simplify electric mobility across regions and manufacturers by ensuring charging infrastructure and vehicle technology compatibility. The review evaluates algorithms and mathematical models that maximize efficiency, reduce costs, and improve charging resource accessibility. Urban planners and policymakers need these optimization strategies to improve EV infrastructure. Article ends with research ideas. It identifies knowledge gaps and suggests research to improve EV technologies and charging systems. To promote electric vehicle adoption and innovation, the recommendations address technical and socioeconomic barriers. Researchers, engineers, and decision-makers use this review to develop and implement electric vehicle technologies and infrastructure.

## 1 Introduction

**Fig. 1:**
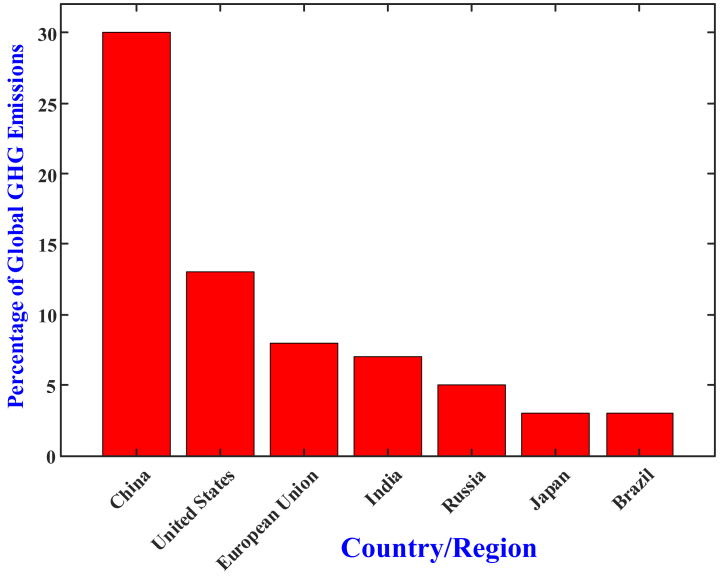
Greenhouse gas emissions by country/Region.

Over the span of the last ten years, the technology behind electric vehicles (EVs) has undergone a period of extraordinary expansion due to the numerous benefits that it provides. Mostly from road travel, the transportation sector accounts for approximately fourteen percent of world greenhouse gas (GHG) emissions. China, the largest emitter in the world, generates roughly thirty percent of all emissions from coal power and heavy industry mostly. While emissions have surged with industrialization, China wants to reduce emissions by 2030 and achieve carbon neutrality by 2060. Currently ranking second among all emitter, the United States accounts for 13–15 % of emissions mostly from transportation and electricity generation. By 2030 it aims to reach net-zero by 2025 and cut emissions by 50–52 %. With about 8 % of world emissions, the European Union has made notable progress by changing to renewable energy and regulating industry emissions. The EU aims a 55 % emissions cut by 2030 and carbon neutrality by 2050. Third-largest emitter at 7 %, India suffers rising emissions from economic development and mostly depends on coal for power. Still, India, stressing renewable energy, has a target net-zero emissions by 2070 [1]. Industry requires a cleaner substitute such as electric vehicles (EVs), which can reduce environmental impact, boost energy efficiency, and reduce dependency on fossil fuels. EVs greatly lower carbon footprints and help to improve metropolitan air quality since they emit zero tailpipe emissions. Widespread EV adoption by 2025 could reduce world CO2 emissions annually by 1.5 billion metric tons. Unlike internal combustion engine (ICE) vehicles, in which only 20–30 % of grid electricity is used, EVs convert 60–70 % of it into movement, so increasing their energy efficiency. Despite being charged from coal fuel plants, EVs emit less than ICE vehicles. EVs help to reduce dependency on limited fossil fuels including diesel and gasoline. Renewable energy can propel them, enhancing energy security and supporting the global movement away from gasoline. EVs are capable of relying on renewable energy sources for its charging, that bring the energy security for future. Also, the EV can stabilize the grid during the peak operating time using Vehicle to Grid Technology. Also the Government of various nations bring policies in terms of incentives, relaxation on taxes to encourage the people to use EV [1]. Electric vehicles are becoming an increasingly important part of the world's transportation system as nations work toward achieving net-zero emissions. In order to overcome the challenges that are currently being faced and to satisfy the growing demand for transportation solutions that are cleaner and more sustainable, it is necessary to conduct research on batteries, charging infrastructure, and energy management systems. This research need to be carried out (see Fig. 1).

**Fig. 2:**
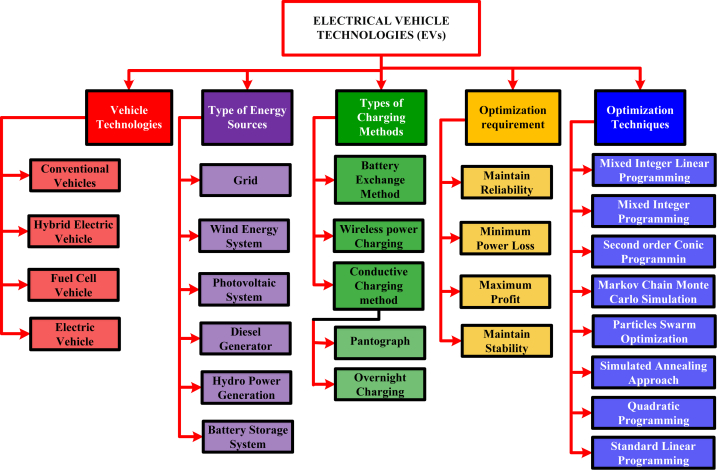
The overall outline of this review article.

China planned to implement 180 GW of wind energy conversion systems and 20 GW of Solar Photovoltaic systems before 2020 [2,3]. The US government is planning to replace the solar PV system, for the energy demand. The aim is to generate 30 % of the energy demand from solar photovoltaic systems before 2030 [4,5]. The recent developments in the EVs technologies enhanced the social benefits and economic development in industrial applications and transportation applications. The main limitation of the EVs technology is the usage of the storage units like a battery. The selection of the battery has certain limitations like battery capacity, size, charging/discharging rate, weight, dimensions, and cost. These limitations increase the cost of the EV and limit the size of the EV. Many researchers are working to optimize the limitations that arise in battery sizing [6,7]. Many automobile industries and countries were investing more money in advanced battery technologies for EVs. The aim is to design a battery with a greater life span, less cost, small in size high storage capacity, and so on. The US government spends two billion dollars for the development of batteries in EV and also, the Google spends ten million dollars for the same [8,9]. The US government planned to implement one million EV charging stations on the roads in the next five years. Australian Energy Market Commission (AEMC) takes a survey in the year of 2019, and as per the survey, the sales of EV are increased in recent years, where in 2019 the number of EVs sold is 203 % higher than the number of sales in 2018 [10]. New Zealand planned to replace electricity generation with renewable energy resources before 20235. The total energy consumption of New Zealand from various sources has shown in Fig. 2. They depend on 15 % of fossil fuels, whereas in the percentage, around 30 % of the power generation from fossil fuels is used for the transportation sectors. The replacement of transportation with electrical vehicles will reduce the consumption of fossil fuels [11].

It is estimated that the vast majority of automobiles in the modern era are powered by internal combustion engines. These engines are responsible for the emission of a considerable quantity of carbon dioxide (CO2), which contributes to the acceleration of climate change. Electric vehicles (EVs) are vehicles that run on electric motors rather than combustion engines. This eliminates the need for fuel and results in zero emissions of carbon dioxide (CO2). The transition to electric vehicles (EVs) reduces emissions and is in line with the goals of environmental protection. Increasing numbers of electrical industries are making the switch to renewable power sources like solar photovoltaics and wind energy in order to reduce the negative impact that they have on the environment with regard to climate change. By the year 2020, China intends to have installed 180 GW (GW) of wind energy conversion systems and 20 GW (GW) of solar photovoltaic systems [2,3]. The United States of America has set a goal to meet thirty percent of its energy requirements through the utilization of solar photovoltaic systems by the year 2030 [4,5], which is in line with the global movement toward the generation of sustainable energy. There have been significant advancements in the areas of environmental conservation, societal well-being, and the development of the industrial and transportation sectors as a result of the advancements in electric vehicle (EV) technologies. Both the performance and the cost of battery storage units are factors that are preventing the widespread adoption of electric vehicles. Electric vehicles (EVs) are dependent on these batteries; however, the development of these batteries is limited by a number of factors, including the capacity of the battery, its size, the rate at which it charges and discharges, its weight, its dimensions, and its cost. Consequently, the prices of electric vehicles go up as a result of these limitations, which also restrict the design and practicability of these vehicles.

The optimization of battery technology is currently the subject of a significant amount of research to which generous funding is being provided. Numerous automobile manufacturers and governments are making significant investments in the research and development of battery technologies that are low-cost, compact, and have a high capacity. Google has made an investment of ten million dollars, while the United States government has committed two billion dollars to the research and development of electric vehicle (EV) battery technology [8,9]. Over the course of the next five years, the government of the United States of America plans to install one million charging stations for electric vehicles (EVs) across the country. This development is part of the infrastructure development initiative. The goal of this initiative is to make room for the growing number of electric vehicles. The Australian Energy Market Commission (AEMC) reported that electric vehicle sales increased by 203 % in 2019 compared to the previous year, which suggests that there is an increase in consumer interest and market growth [10]. As of the year 2035, New Zealand has committed to reducing its reliance on fossil fuels as a primary source of energy. Renewable energy sources will be used in place of traditional sources of electricity generation in order to accomplish this goal. Transportation accounts for thirty percent of New Zealand's total energy consumption [11], and fossil fuels are responsible for fifteen percent of the country's energy supply [12]. The transition to electric vehicles is a demonstration of the nation's intention to reduce its reliance on fossil fuels. In order to achieve sustainability, protect the environment, and adopt technologies that are cleaner and more environmentally friendly, this transition in energy sourcing is an essential step that must be taken. Tax rebates, grants, and the privilege of accessing high-occupancy vehicle lanes are all factors that contribute to the increased adoption of electric vehicles (EVs) [13]. An increase in consumer interest is a direct result of these incentives, which also contribute to the growth of the market. Electric vehicles (EVs) are becoming more appealing to a wider demographic as a result of advancements in advanced regenerative braking systems and aerodynamics. These advancements are increasing the efficiency of EVs as well as their range. As a result of these advancements, it is anticipated that the growth of the low-carbon economy will be accelerated, and that global transportation will undergo a transformation that is both more sustainable and innovative.In this work, a short review has been presented about the EV technology with its corresponding details likes, like charging methods and standards, charging stations, and optimization methods for the implementation of the EV charging station.

Highlighting the most recent developments and trends, this research article will provide a complete summary of improvements in electric vehicle (EV) technologies. As it analyzes their respective advantages, disadvantages, and relationship with renewable energy sources, it will classify several EV charging methods including conductive, wireless, and battery exchange approaches. Moreover covered in the paper will be optimization strategies meant to improve grid stability, reduce costs, and maximize EV charging efficiency. At last, it will show how environmentally friendly development advances EV technologies and infrastructure to support global green energy projects. The ouline of this research article is shown in Fig. 2.

## 2 Vehicle technologies

For more than two hundred years, fossil fuels have been the primary source of energy for transportation, which has resulted in significant emissions of carbon dioxide and an increase in the cost of fuel. In addition to reducing emissions of carbon dioxide (CO2), electric vehicles (EVs) also reduce the amount of money spent on fuel, making them an extremely encouraging solution. Because of the gradual depletion of fossil fuel reserves, it is becoming increasingly important to make use of alternative energy sources. This highlights the significance of technological advancements in the field of automobile advancement. Electric vehicles, hybrid electric vehicles, fuel cell vehicles, and conventional vehicles are the four primary categories of vehicles that have made the transition from conventional to advanced [14]. Vehicles that are considered conventional are those that run on fossil fuels and produce a significant amount of carbon emissions. The combustion engine and the battery are both utilized in the operation of transitional hybrid electric vehicles. The vehicle in question is equipped with a dual-source system, which enables it to make use of either the battery or the combustion engine, or both. As a result, it produces lower levels of carbon dioxide emissions in comparison to conventional automobiles.

**Fig. 3:**
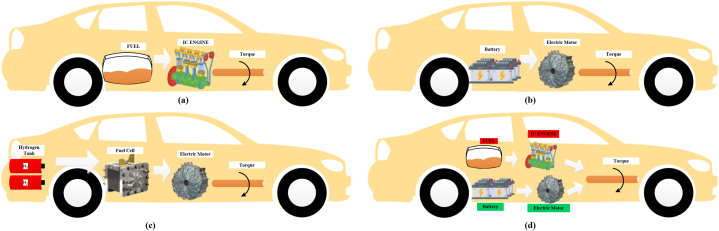
Various electrification methods in the transportation system.

Both fuel cell vehicles and electric vehicles have the potential to significantly reduce carbon emissions by making use of renewable energy sources. This will result in emissions levels that are extremely close to zero. Fuel cell vehicles are vehicles that generate electricity through the use of hydrogen, which is then used to power an electric motor. Due to the fact that it only produces heat and water vapor, it is a source of energy that is friendly to the environment. Electric vehicles that are powered by batteries, on the other hand, are completely powered by electricity. Electrically powered automobiles that do not emit any emissions from their exhaust pipes are the epitome of the concept of electrification. As a result of advancements in battery technology, electric vehicles (EVs) are becoming more and more feasible for use in everyday life. This is primarily due to the fact that their range has increased and they are able to charge more quickly. The second category of electric vehicles is comprised of hybrid electric vehicles, which provide a versatile combination of the benefits that are associated with both internal combustion engines and electric motors. The process of hybridization is able to effectively reduce the amount of fuel that is consumed and the amount of harmful emissions that are released in urban traffic conditions that are filled with congestion. Due to the fact that the vehicle switches between the internal combustion engine and electric power depending on the driving conditions, it is able to conserve energy and reduce emissions during the driving process. The various ways in which these automobiles can be powered by electricity are depicted illustrated in Fig. 3.

In rural areas where there is a lack of charging infrastructure, hybrid electric vehicles (HEVs) are advantageous because of their ability to charge. In the event that the battery runs out of power, the vehicle is able to switch over to the internal combustion engine in a seamless manner, which enables the vehicle to continue operating without the need for an electrical charging station. Furthermore, regenerative braking is utilized in order to replenish the charge of the battery while the vehicle is decelerating, which ultimately results in an increase in both efficiency and range. At this point in time, electric vehicles (EVs) are in the preliminary stages of development, and it is anticipated that it will take approximately ten more years for them to become widely adopted. In the midst of this period of transition, hybrid electric vehicles are ideal because they are both practical and efficient [[14], [15], [16]]. They combine the efficiency of electric propulsion with the dependability of internal combustion engines to create a comprehensive solution. It is possible to improve the efficiency of hybrid electric vehicles by carefully selecting the specifications for both the internal combustion engine and the electric motor. These vehicles have a number of advantages over conventional ones, and those advantages should not be overlooked. The ability of these vehicles to switch between different sources of energy in response to the conditions of the road results in a reduction in the amount of fuel that they consume initially [17]. Additionally, when compared to vehicles that are powered solely by internal combustion engines, hybrid electric vehicles (HEVs) produce a lower amount of carbon emissions. The energy efficiency of vehicles can be improved through the use of regenerative braking to charge their batteries.

**Fig. 4:**
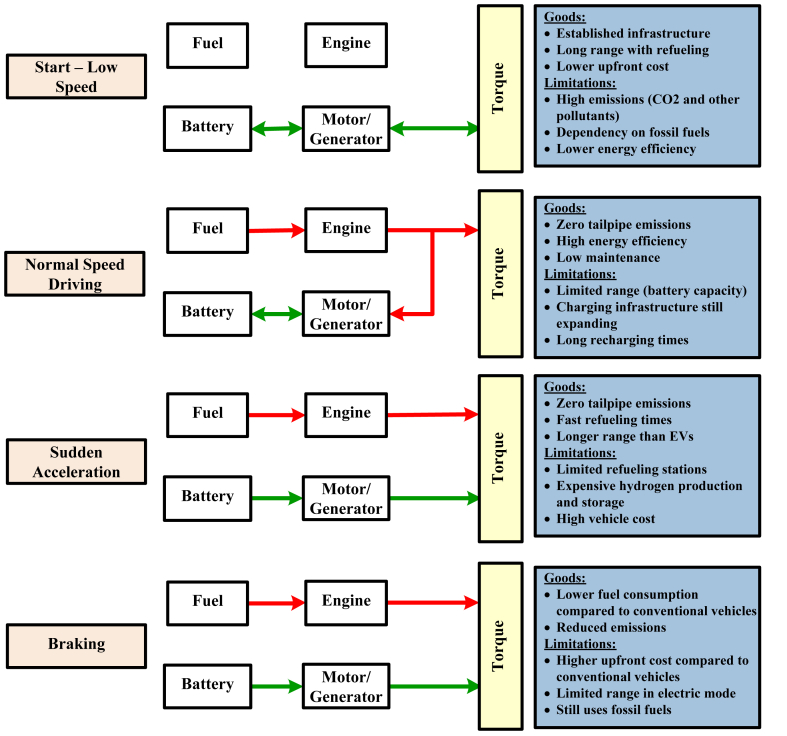
Different modes of operation in Hybrid EV.

When it comes to transferring power from the battery or the combustion engine to the wheels, the drivetrain system of a hybrid electric vehicle functions as an extremely important component. Drivetrains that are hybrid in nature are utilized [18,19]. These hybrid drivetrains include series-hybrid (SH), parallel-hybrid (PH), and series-parallel (SP). In order to supply the vehicle with power, the SH drivetrain system can either make use of the engine or the battery simultaneously. When a vehicle is primarily powered by batteries but switches to the engine once the battery has been depleted, this configuration is advantageous because it allows the vehicle to continue to function.The PH drivetrain system is able to achieve a harmonious balance between power and efficiency by making efficient use of both the engine and the battery in conjunction with one another. The fact that this system is able to effectively combine both types of energy sources makes it particularly well-suited for high-power scenarios. The series-parallel (SP) drivetrain system offers the greatest degree of versatility because it enables the vehicle to function on either the engine or the battery independently or simultaneously. The purpose of this hybrid system is to effectively regulate power in a variety of driving conditions by combining the benefits and drawbacks of both series and parallel systems.The benefits that each system offers are unique, and they are designed to accommodate a wide range of driving styles and requirements. The research [20] sheds light on the complexity and functionality of these systems, and the operational mechanics of these systems are illustrated in functional diagrams such as Fig. 4. By effectively regulating the distribution of power between their two sources, hybrid vehicles are able to improve their efficiency and reduce the amount of harmful substances that are released into the environment. This comprehensive diagram illustrates how hybrid vehicles accomplish these improvements.

In order to improve both their performance and their energy efficiency, hybrid electric vehicles (HEVs) make use of a complex drivetrain system that integrates series and parallel components. This controller, which also functions as the central processing unit of the vehicle, is responsible for regulating the operation of the power distribution circuits, as well as the engine and the battery. The controller's primary responsibility is to ensure that the dynamics of the vehicle are properly monitored and managed. The motor, generator, vehicle, and braking velocities are some of the essential variables that are quantified by this software. In addition, it makes a measurement of the torque that is produced by both the generator and the motor. On the basis of these inputs, the controller makes adjustments to the flow of power between the engine and the battery in order to maximize the efficiency with which energy is distributed in order to meet the operational requirements of the vehicle. The controller may make use of additional battery power in order to improve the performance of the engine while minimizing the amount of fuel that is wasted during the process of increasing the speed of the vehicle.

A key controller is responsible for overseeing the process of charging and discharging the battery, which is an important role that they play. For the purpose of preserving the health of the battery and maximizing the range and efficiency of the vehicle, effective management is essential. The controller is vital to the process of regenerative braking because it is responsible for converting the kinetic energy, which would otherwise be lost as heat, into electrical energy. For the purpose of transferring energy from the braking process to the battery, this process makes use of the motor as a generator. Regenerative braking in hybrid systems improves energy efficiency and extends range by reclaiming energy that would otherwise be lost in conventional vehicles [21,22]. This allows hybrid systems to achieve greater range and energy efficiency. In addition to this, the series-parallel drivetrain is able to provide a smooth transition between the utilization of power from the engine and the battery itself. The adaptability of the vehicle enables it to function with either electric power, power from an internal combustion engine, or a combination of the two different types of power. The mode is selected in a dynamic manner based on the driving conditions, the inputs from the driver, and the charge of the battery. The series-parallel drivetrain system is an example of sophisticated hybrid engineering, which effectively reduces the amount of fuel consumed and emissions produced by the vehicle while simultaneously maintaining or improving its performance. These technologies are a prime example of the automotive industry's commitment to innovation and environmental responsibility. They make it possible to develop transportation options that are less harmful to the environment.

When it comes to hybrid electric vehicles (HEVs), the motor, generator, DC-DC converters, and batteries make up the various components that make up the electrical systems. The vehicle's ability to transition between electric and combustion power sources in a seamless manner is made possible by these components, which contribute to the vehicle's overall efficiency. By making use of a throttle position sensor, the controller is able to control the amount of fuel that is delivered to the combustion engine. To achieve the highest possible fuel efficiency while simultaneously reducing emissions, it is necessary to implement control measures with great attention to detail.The system is managed by the controller in order to maximize the utilization of the available resources. However, depending on the particular driving conditions, the vehicle can function by utilizing either the engine or the battery, or by utilizing a combination of the two. Quantification of variables such as velocity, braking force, acceleration, road gradient, and wind drag is something that the dynamics system of the vehicle is responsible for. The performance of the vehicle is significantly influenced by these parameters, which are continuously monitored in order to make any necessary adjustments to the vehicle's operation.

Depending on the speed at which the vehicle is traveling, hybrid vehicles can operate in one of four distinct modes: low to medium speed, normal speed, sudden acceleration, and braking [23]. Various driving conditions are illustrates the manner in which each mode maximizes efficiency and enhances performance. Electric vehicles (EVs), on the other hand, improve hybrid technology and overcome a number of the limitations that these technologies have. Electric vehicles (EVs) are experiencing a continuous expansion of their capabilities as a result of ongoing advancements in research [24]. The vehicle dynamics system in electric vehicles (EVs) is responsible for monitoring parameters such as speed, inclination, and the State of Charge (SoC) of the battery in order to effectively manage electric vehicle energy consumption.

Electric vehicles are solely dependent on battery storage, which completely eliminates the emission of pollutants from the exhaust pipe and significantly reduces the environmental footprint that these vehicles leave behind. The battery supplies power to the motor while the vehicle is moving forward at a high rate of speed. In the opposite direction, the regenerative braking system is responsible for recharging the battery whenever the vehicle is applying the brakes. This function maximizes the amount of energy that is reclaimed and increases the driving range of vehicles in urban areas that require frequent stops. Two primary modes of operation are available for electric vehicles (EVs), which are acceleration and deceleration/braking. A demonstration of how the energy consumption and performance of the vehicle can be optimized for each mode. In the acceleration mode, the battery power is channeled to the motor in the most efficient manner possible, whereas in the deceleration/braking mode, the energy recuperation is maximized through the utilization of regenerative braking. Electric vehicles (EVs) are able to improve their efficiency by operating in dual-mode, which makes them an excellent choice for applications that take place in urban scenarios. By acting in this manner, they have the potential to effectively reduce carbon emissions and make a contribution to the reduction of global warming risks. Another vehicle technology is a fuel cell electric vehicle, which is used for a low-speed and smooth operation like buses, elevators, etc [25]. Various automobile industries and companies like Toyota, and Hyundai were producing highly reliable fuel-cell electric vehicles in recent years. The efficiency and economy of fuel-cell electric vehicles have been obtained by several energy management methods [26]. The Fuel cell hybrid electric vehicles are another kind of vehicle technology, where the combination of fuel cell with power management and battery bank, ultra-capacitors with power management method has been used. The battery can be charged or discharged with respect to power generation and power demand. The fuelcell is beenconsidered the most important energy source for powering electric vehicles and the battery storage system/ultra-capacitors are supporting the vehicle for stable operation [[27], [28]]. The main drawback of this work is the size of the fuel cell and battery.

## 3 Types of batteries

**Fig. 5:**
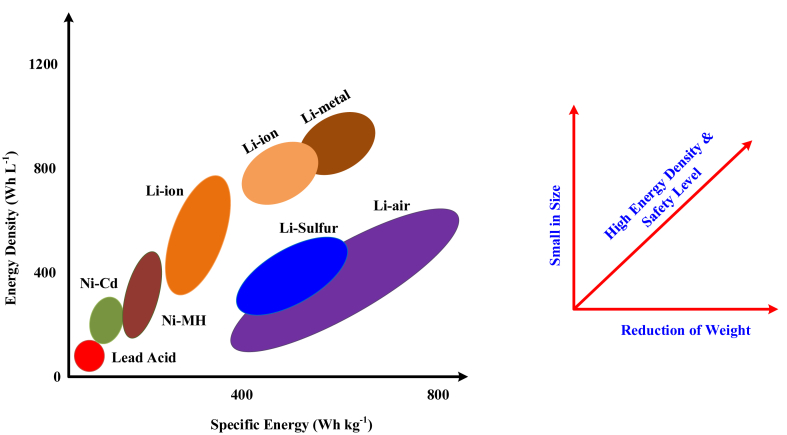
Characteristics of various batteries and its energy density.

Batteries are the heart of electric vehicles, storing electrical energy that powers the motor [6]. There are different types of batteries are available in the commercial market and each have certain characteristics with respect to the type of applications. The evolution of batteries and its impact is shown in Fig. 5. The different kind of batteries and its characteristics are given in Table 1.

**Table 1:** Types of batteries and its characteristics.

Types of Batteries	Advantages	Limitations
Nickel-Cadmium (NiCd) Batteries	• High robustness, • Long life cycle, • Good performance across a wide range of temperatures. • Ability to deliver high surge currents	• Environmental and health concerns due to cadmium, a toxic heavy metal. • Suffers from a memory effect where batteries lose maximum capacity if not fully discharged before recharging. • Lower energy density
Lithium-Ion Batteries	• Batteries with high energy density are lighter and have a greater amount of energy stored in them. • They can be charged more quickly compared to other types of batteries. • These batteries are extensively used in current electric vehicle models because of their well-developed technology.	• Electric vehicle prices are increased by high costs. • Hazards associated with thermal runaway (excessive heat) and fires. • Performance can be reduced by extreme temperatures. • Requires sophisticated battery management capabilities.
Lithium-Sulfur (Li-S) Batteries	• Increased theoretical energy density has the potential to increase the distance that vehicles can travel. • Sulfur is more cost-effective and readily available compared to cobalt, which has the potential to reduce expenses.	• Limited number of charge cycles and reduced capacity after a few recharges. • Challenges related to the dissolution of lithium polysulfide and the expansion of sulfur volume. • Still on developing solutions that enhance the durability and efficiency of commercial products.
Lithium Iron Phosphate (LiFePO4) Batteries	• Improved safety due to decreased likelihood of thermal runaway. • A long cycle life reduces the need for frequent battery replacements. • Suitable for electric vehicles that prioritize safety and durability rather than energy density.	• Lower energy density results in batteries that are more weighty and larger in size. • The efficiency and range of these batteries are limited when compared to other lithium-ion batteries. • Performance can be affected by cold temperatures, which restricts their use in colder regions.

### 3.1 Battery management systems (BMS) in electric vehicles (EVs)

The BMS is act as the brain of electric vehicles, is responsible for controlling the battery pack. The modern architecture of electric vehicles is dependent on them for the safety, reliability, performance, and longevity of the battery cells. BMS prevent battery cells from being overcharged and from being deeply discharged for safety reasons. Through the monitoring of cell voltage and temperature, it is possible to prevent explosions and fires caused by thermal runaway in batteries. By incorporating cooling systems, the BMS helps to ensure that the battery pack does not overheat, thereby enhancing the safety of the vehicle. The balancing of cells is yet another essential function of the BMS. Packing battery cells that have slightly different capacities and resistances can result in charging and discharging power that is not distributed evenly. Because of this discrepancy, the efficiency and lifespan of the battery pack may be reduced. The BMS maintains the uniformity and efficiency of the battery pack by balancing the cells in a passive (resistive) and active (energy transfer between cells) manner.

In addition to ensuring safety, a BMS improves performance. It determines the battery's State of Charge (SoC), which is the percentage of charge that is still available, as well as its State of Health (SoH), which is the battery's overall condition and its useful life. The range of electric vehicles is predicted, energy consumption is managed, and maintenance and battery replacement are scheduled. The power output of a BMS is adjusted based on the current state in order to improve both performance and the life of the battery. This dynamic management optimizes the energy efficiency of the battery by adjusting the charging and discharging cycles by taking into account the current usage and the conditions of the environment. BMSs are necessary for ensuring both consumer confidence and regulatory compliance. In order to ensure the safety, efficiency, and performance of a vehicle, a BMS is required by a number of safety and performance standards. In order to build consumer trust in electric vehicle technology, particularly in terms of range and battery life, a dependable BMS system is required. For the purpose of enhancing performance, advanced machine learning algorithms should be used to forecast the life of batteries and optimize energy management strategies in BMS technology devices. The integration of vehicle telematics could provide drivers and owners with useful information such as real-time diagnostics and alerts for predictive maintenance. As the Internet of Things (IoT) continues to expand, BMS may be able to provide cloud-based analytics, remote firmware updates, and integration with smart grids and home energy systems in order to provide advanced energy management solutions.

### 3.2 Different types of charging methods

**Fig. 6:**
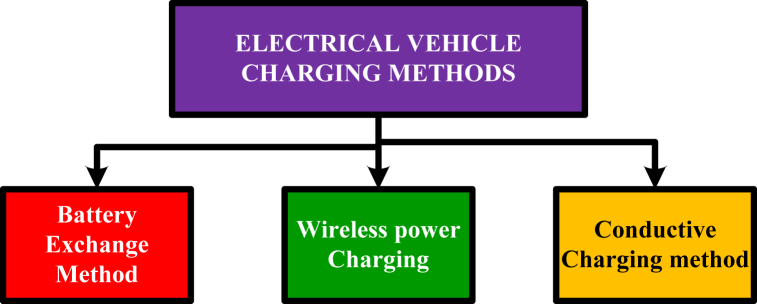
Electrical vehicle charging methods.

The charging methods are different with respect the vehicle technologies. There are different kinds of charging methods are used for charging vehicles. It is very necessary to understand the charging methods for doing research on electrical vehicles. There are three different charging techniques are used in the EV field and the techniques are the battery exchange method, conductive charging method, and wireless charging method as shown in Fig. 6. The conductive charging method has been divided into two types pantograph charging and overnight depot charging.

The battery exchange method is also known as the battery swapping method. In this method, the battery has been replaced or exchanged in the battery exchanging station, where the exhausted battery is replaced with the charged method and when the new battery exhausting then the older battery has been replaced with charge. For one charge, the consumer must pay the money to the battery exchanging station. In this method, the exhausted battery has been charged slowly for the next replacement. This method enhances the battery life because of the slow charging [28]. Consumers need not wait for a long time for the battery charging, the battery exchange will take only a few minutes. Another advantage of this kind of battery charging method is, the battery can be charged by renewable power generation as the charging station generates power using renewable energy sources. So that, the carbon emission on the vehicle can be eliminated [[29], [30]]. There are some disadvantages to this kind of battery charging, and they are, in high traffic areas it will be not suitable, its cost is quite expensive as compared to other methods, the unavailability of the batteries in some charging due to the vehicle standard and battery specification [31,32].

**Fig. 7:**
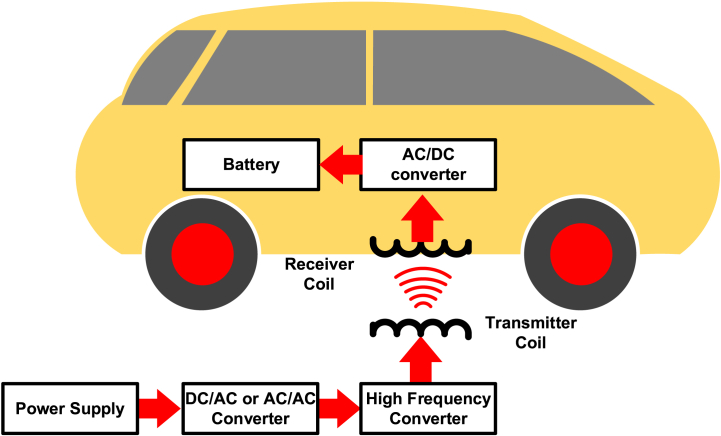
Electrical vehicle charging methods.

**Fig. 8:**
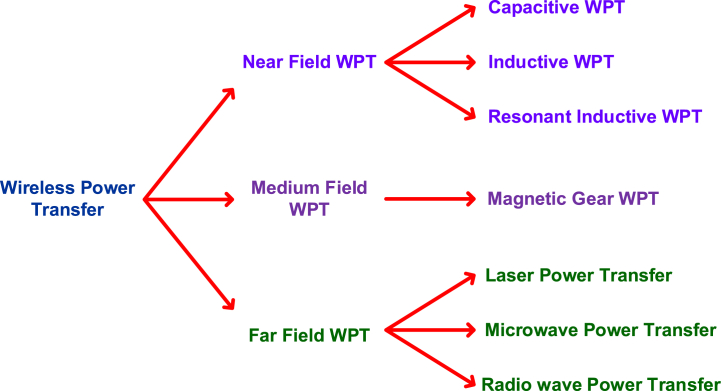
Different types of wireless power transfer methods.

Wireless battery charging technology is the second method for charging electric vehicles. This method uses electromagnetic induction as shown in Fig. 7. In this method, the coils are used for creating the electromagnetic field. There are two coils primary coil and secondary coil. The primary coil is placed on the roadsides, and the secondary coils are placed in the cars. In recent times, wireless battery charging gains its fame in the energy market, because of its convenient and safe recharge method. The vehicle need not be rested for charging. This method can charge the battery in the vehicle running condition. Also, there is no limitation with respect to the battery standard, vehicle model, and other parameters. Based on the secondary coil's specification, this method charges the battery [33]. The basic operation of wireless battery charging is electromagnetic induction, whereas this inductive power is not efficient for power transfer. This kind of charging will be efficient when the distance between two coils is between 20 cm and 100 cm [34]. And another factor limiting the efficiency of this wireless transfer is eddy current. Also, the communication network is affected by electromagnetic waves and vice versa [35]. There are different types of WPT methods are available based on the materials, and distance. It can be classified into three categories such as near field WPT, Medium field WPT and far field WPTNear Field WPTtransfers the power between the short ranges of distance like millimeters to centimeters. This type of WPT is more efficient as compared to other methods. There are further classifications are in the near field WPTsuch as capacitive, inductive and resonant inductive WPT as shown in Fig. 8. Capacitive WPT uses two conducting plates for transferring power from one plate to another plate. One of the plates will be placed on the source side and another one will be placed at the application side. This kind of WPT is useful for the small devices like mobile phones, medical equipment, and so on.Inductive WPT uses the electromagnetic induction principle to transfer power from one coil to another coil. The coil at the source side will be energized and the magnetic flux lines will be conducted by the coil implemented at the application side. This method is useful for all kinds of applications such as mobile phones, medical equipment, EVs andetc.Resonant Inductive WPT applies the resonance in inductive power transfer method. This tunes both transmitter and receiver coils to be operated on the resonance frequency. This improves the efficiency WPT. This method can be useful for the EV charging applications and more efficient. The dynamic transmitter coil used at the charging station in order to adapt the frequency of various types of receiver coils at the vehicle side. Medium Field WPT uses the magnetic field for transferring power using intermediate poles. The alignment of the of the intermediate coils (gears) should be straight for the efficient transfer. In far field WPT, the waves have been used such as laser rays, microwaves, and radio waves. These laser power transfer, the laser light has been projected to the photovoltaic panels of the receiver side, and these panels produce electricity. These methods are not efficient as compared to other two methods; however it will be suitable for the applications such as remote applications, military applications, and space research applications [34,35].

This method is like plugin the battery directly with the source for the recharging. The vehicle has a plugin port, with a power conditioning unit. When this plugin is connected to the supply, the battery can get charged. When the plugin is disconnected the charging state will be cut. This method has different charging levels based on the vehicle standard and battery specification. Generally, it was classified as L1 charging, L2 charging, and L3 charging, where the L stands for levels. It is possible to transfer power from the battery to the grid in the conductive method. So, this method has less power loss during the charging, and also, the voltage level and reactive power compensation on the battery can be maintained [36,37]. The L3 charging method has differed from the other levels of charging, and this creates a voltage deviation in the distribution system. The power deviation affects the reliability of the system, which increases the power loss and reduces the life span of the battery and charging units [38,39]. This method requires a complex infrastructure and has a limitation on the power grid [40]. The vehicle to grid technology requires proper communication between the vehicle and the grid. Also, it requires frequent charging, which reduces the battery's health. In heavy load applications like buses, it requires a high battery capacity, which needs more charging time. There are quick charging methods are developed for charging heavy-load applications [[41], [42], [43], [44]]. Two different types of charging methods like overnight depot charging method and the pantograph charging method were used for charging heavy-load applications. The overnight depot charging method is a simple and effective method, which allows slow charging on the battery, and the charging can be completed during the startup time. This method is highly recommended for the heavy load application, where more amount has been spent on the battery as the initial cost. So it is mandatory to provide the proper maintenance to the battery. This overnight charging method directly increases the battery life span. The pantograph charging provides the less investment method for charging the higher load applications. But the infrastructure for implementing this charging method requires more cost [43]. This pantograph charging method has been divided into two more categories such as a top-down pantograph and a bottom-up pantograph.

The Battery Exchange Method offers a number of benefits and drawbacks. One of the main benefits of BEM, claims [31], is its capacity to balance power demand using Vehicle-to- Grid (V2G) and Grid-to- Vehicle (G2V) technologies [45]. Furthermore [32], emphasizes how readily BEM can be combined with renewable energy sources, so offering a flexible solution for sustainable energy integration. As mentioned in Ref. [46], another advantage is the less time needed for battery replacement, which can be crucial for consumers searching for fast charging solutions. Moreover [47], notes that longer battery life made possible by BEM helps the electric vehicle to perform generally. BEM does, nevertheless, also have certain clear drawbacks. For example [31], notes that maintaining stocks is quite difficult since spare batteries must be easily accessible to support the exchange mechanism. Furthermore discussed in Ref. [32] are the difficulty of preserving a wide range of battery specifications and the necessity of more area to suit the exchange infrastructure. Another restriction is the great expense related with BEM since [46] shows that the approach can be expensive. Likewise [47], underlines that the first outlay for batteries and associated equipment is rather high, which could discourage acceptance. Advantages and drawbacks abound in the Wireless Power Charging (WPC) approach as well. As said in Ref. [35], one of the main benefits is that recharging can be done while the car is in motion, so saving user convenience and minimizing downtime. Furthermore, emphasizes how easy WPC is, especially since it does away with the need for any conductors, so simplifying and user-friendliness of the process. WPC has several drawbacks, too. Effective recharging depends on the real-time flow of power between the transmitter and the receiver, thus any misalignment can disturb the process [35]. Furthermore, notes that the efficiency of power transfer is constrained to a particular distance range, usually between 20 cm and 100 cm, which can so limit its relevance in some situations. Another approach in the literature is conductive charging. As noted in Ref. [36], one of its main benefits is that it supports several levels of charging, so enabling flexibility depending on user need [37]. also notes it as a quick charging technique with consistent energy transfer. Emphasized in Ref. [38] is another main benefit: vehicle-to---grid (V2G) technology lets electric vehicles interact with the grid in both directions. As shown in Ref. [39], conductive charging is also helpful in lowering power losses; whereas [40], notes that, under appropriate control, it can help prevent grid overload. Moreover [46], points out that this approach allows one to compensate reactive power, so supporting grid stability. However, conductive charging has certain disadvantages [36]. The infrastructure needed for conductive charging can be complicated, thus deployment may prove more difficult. Furthermore limiting the adaptability of V2G technology is [37]'s observation that the grid limits bidirectional power flow. The fast speed of technological changes is discussed in Ref. [38] as a cause of worry since it might influence system voltage stability. As emphasized in Ref. [39] conductive charging also depends on careful charge monitoring; improper control of charging and discharging can cause grid overloading, as noted in Ref. [40]. At last [46], notes that frequent charging and discharging cycles of V2G technology can shorten battery lifetime even if it can be advantageous.

### 3.3 V2G challenges

Vehicle-to- Grid (V2G) technology provides ancillary services including frequency control by means of bidirectional energy flow between electric vehicles (EVs) and the grid, and lets EV discharge power back to the grid during times of great demand. Still, V2G offers certain technical difficulties [[48], [49], [50], [51]].

• Regular charging and discharging cycles could accelerate battery degradation, so affecting EV lifetime and performance. Research on battery management systems (BMS) is still under progress to help to solve this problem.
• V2G asks for specific bidirectional chargers capable of running the vehicle and releasing energy back to the grid. Less often found and more costly than traditional unidirectional chargers are these ones.
• Good V2G deployment depends on grid infrastructure able of regulating dynamic power flows, which might demand major grid upgrades from the current situation.
• Standardized communication protocols like ISO 15118 help to enable smooth integration of EVs into the grid; still, interoperability remains difficult.

### 3.4 EV charging standards

The several international charging standards reflect the several regional needs, regulatory environments, and historical market developments. The various standards associated with the charging stations are listed in Table 2. Still, the lack of a clear global benchmark creates significant challenges [[52], [53], [54]].

• Every area now has a standard connector type, voltage range, and power rating unique to that area. China has developed its own GB/T standard, for instance; Europe has chosen the Type 2 connector as the default; North America uses the J1772 (Type 1) for AC charging. These regional variations make it difficult to create a global benchmark satisfying every interested party.
• Unless multi-standard chargers are used, which increases infrastructure costs, vehicles meant for one area could not be compatible with charging infrastructure in another. This becomes a more major issue for EV drivers flying abroad and needing several adapters.
• Different communication techniques followed by different standards complicate the integration of smart charging and Vehicle-to- Grid (V2G) capabilities. For instance, the CHAdeMO standard supports bi-directional communication (V2G), but not all standards are created to sufficiently satisfy such needs.
• Globally standardizing of charging infrastructure is challenging since different nations have regulating systems for grid integration, safety, and power distribution. Harmonizing regulations across many sectors is a difficult work needing government collaboration and cross-industry involvement.
• Industry Resistance: Since every company has heavily spent in developing technologies around current regional standards, automakers and infrastructure providers could be reluctant to embrace a single standard. Using a uniform standard would mean retrofitting present charging stations and retooling manufacturing processes.

**Table 2:** Comparison of international EV charging standards.

Standard	Region	Connector Types	Maximum Power Output	Voltage Levels	Communication Protocols	Key Features
SAE J1772 (Type 1)	North America	Type 1 (J1772)	AC: 19.2 kW (Level 2)	120–240V (AC)	Pilot signal (PWM), PLC	Widely used for AC charging in the U.S. and Canada.
IEC 62196 (Type 2)	Europe, Australia	Type 2 (Mennekes)	AC: 43 kW, DC: 350 kW	230–400V (AC), 400–1000V (DC)	PLC, ISO 15118	Standard in Europe for both AC and DC charging.
CHAdeMO	Japan, Global	CHAdeMO	DC: 62.5 kW (up to 400 kW)	Up to 1000V (DC)	CAN bus	Primarily for DC fast charging, bi-directional support.
GB/T	China	GB/T	DC: 237.5 kW (up to 900 kW)	450–1000V (DC)	Proprietary (GBT 20234)	China's national standard for AC/DC charging.
CCS (Combo 1 & 2)	Global	Combo 1 (SAE), Combo 2 (IEC)	DC: 350 kW	Up to 1000V (DC)	PLC, ISO 15118	Widely adopted in Europe and North America for fast charging.

Since global standardizing of EV charging infrastructure is still difficult, projects aiming at interoperability and the creation of multi-standard chargers help to close local gaps. Further enabling more sensible charging solutions is development in wireless charging standards including SAE J2954 and IEC 61980. Effective global EV adoption to scale depends on harmonized solutions that satisfy the various needs of different markets so enabling constant cooperation among manufacturers, infrastructure builders, and regulatory authorities.

## 4 EV charge scheduling process

Optimization is the technique of, within an identified set of limitations, making a system, architecture, or decision as efficient as practically feasible by either minimizing or maximizing a particular objective. Optimization is quite important in the framework of electric vehicles (EVs) for properly and dependably managing several elements, including charging infrastructure, energy consumption, battery management, and grid integration. Several factors define the complicated optimal process in electric vehicle systems: travel paths, charging times, energy costs, and the environmental impact of integration of renewable energy sources. Optimization strategies are absolutely necessary to guarantee that the vehicles satisfy user expectations while reducing costs, delays, and disturbance of the power grid as electric vehicles (EVs) are being embraced more and more [55].

The transportation sector accounts for significant worldwide greenhouse gas emissions, thus reducing these emissions becomes even more important than it is in view from climate change. Electric vehicles (EVs) are cleaner and more sustainable than internal combustion engine driven conventional vehicles. By not producing direct emissions unlike conventional cars, EVs greatly enhance urban air quality and so reduce the global carbon footprint [[56], [57]]. Their capacity to run on renewable energy sources like solar and wind power helps to further lower their environmental impact. Given increasing urbanization and the global drive for decarbonization, adoption of EVs is becoming a main strategy for countries to meet their climate targets and reduce reliance on fossil fuels [[58], [59], [60]].

Energy economy wise, EVs also have several advantages over conventional vehicles. Electric motors are by nature more efficient than internal combustion engines since heat losses a lot of energy. EVs are becoming more dependable and reasonably priced over time since their simpler electric drivetrain requires less maintenance. Governments and manufacturers are also significantly supporting EV technologies and infrastructure, so promoting the expansion of a robust charging network and providing incentives to consumers to migrate to electric cars. These factors make EVs not only a green choice but also a sensible, forward-looking answer to meet future transportation needs.

Optimizing electric vehicle systems has several significant advantages that help to improve performance and user experience both separately. These advantages mostly fit sustainability, cost control, grid stability, and efficiency [61].

Increasing energy efficiency is one of primary goals of optimization in EVs. By improving charging strategies and battery management systems, one can lower energy consumption and so save resources. Good route planning systems, for example, ensure that EVs follow the shortest or most energy-efficient paths, so preserving battery life and reducing general energy consumption [62]. Smart charging systems can similarly lower idle times at charging stations by predicting and regulating charging demand. Moreover, much depends on optimization in lowering the running cost of an EV. Time-of- use (ToU) pricing systems let EVs be charged during off-peak hours when rates of electricity are lower. Optimization methods help to arrange charging times so that these cost savings maximize themselves. Moreover, good battery use can help to reduce the frequency of expensive battery replacements, so reducing the total cost of ownership for EV users. As more EVs join the grid, controlling the additional demand becomes quite challenging. Timing EV charging during low demand will help to balance the demand on the grid and so avoid grid overloads during peak hours. By providing extra power during periods of great demand and by smoothing out fluctuations in the output of renewable energy, optimizing vehicle-to-–grid (V2G) technology—which lets EVs discharge electricity back into the grid—helps to support grid stability.

Optimization guarantees that using their EVs results in less delays and less inconveniences for the consumers. Faster charging and a more perfect user experience follow, for example, from optimizing charging schedules and so lowering waiting times at charging stations. Intelligent battery management also helps consumers to extend the life of the battery, so enabling them to go between charging sessions and so reduce the maintenance demand. Including electric vehicles (EVs) with renewable energy systems and ideal charging techniques can significantly reduce the carbon footprint of transportation. Reducing the dependence on fossil fuels depends on optimization as well since maximizing the use of renewable energy for EV charging requires same. Specifically in the transportation sector, achieving long-term climate targets and advancing sustainability depend mostly on this.\

Despite the advantages, there are several challenges and restrictions in the best optimization of electric vehicle systems. These constraints define technical, financial, and infrastructure factors affecting the general success of optimization projects [[63], [64]]. Maximizing EV systems is mostly difficult because of the changing nature of renewable energy sources. Solar and wind power are intermittent and their availability varies daily and with the season. Especially in places mostly dependent on renewable energy, this volatility makes it difficult to guarantee constant charging availability for EVs. Good optimization requires a strong infrastructure for charging stations and grid management both. Plans for large-scale optimization demand advanced sensors, networks of communication, and real-time data analytics systems now under use. This can be challenging especially in places without developed infrastructure. Furthermore difficult and costly to integrate, the combination of EVs with smart grids requires careful coordination between the grid, charging stations, and car. Although optimization can help to save long-term costs, the initial outlay required to set up the necessary infrastructure and technology can be shockingly high [[65], [66], [67], [68]]. Expensive projects aiming at discouraging general adoption are running the necessary optimization software, installing fast-charging stations, and upgrading the grid to manage growing EV presence. Cybersecurity and Data Privacy: Since EV optimization mostly depends on data exchange between charging stations, grid operators, and vehicles, cybersecurity and data privacy become more likely to be targets of attack. Unauthorized access of vehicle data or grid systems can compromise system security generally. Although protecting user privacy and cybersecurity is essential, doing so complicates things even more and costs more for efforts at optimization. Frequent charging and discharging cycles can over time reduce battery performance, so reducing the lifetime of electric cars generally. While intensive charging plans—especially during times of great demand—may hasten battery wear and compromise EV long-term dependability, optimization strategies can only somewhat control battery consumption [[69], [70], [71], [72]].

Many optimization methods have been developed to handle the difficulties with EV charging, battery management, and grid integration. These methods find the most effective answers to challenging optimization issues by means of advanced mathematical and computational methods.

Inspired by the mechanism of natural selection, genetic algorithms (GA) over time convert a population of candidate solutions to discover the best one [73]. Optimizing multi-objective problems—like reducing charging time while maximizing battery lifetime—allows GAs especially great value. Inspired by social behavior of birds, Particle Swarm Optimization (PSO) is a natural approach. Every particle in the swarm stands for a possible reaction; through neighbor learning, the particles constantly get better. For problems like determining the optimal charging schedule needing extensive search area research, PSO is ideal. Ant Colony Optimization (ACO) is another nature-inspired method grounded on ant behavior seeking for food. Other ants follow the most strong pheromone trail to the food source while ants deposit pheromones on the ground. ACO can enable the shortest travel paths or shortest charging times in EV optimization [[74], [75], [76]].

Chaotic Harris Hawk Optimization (CHHO) develops upon the conventional Harris Hawk Optimization method and so increases the exploration capacity of the method by adding chaotic variables. This allows CHHO to avoid local optima and converge faster to the global optimum, so lowering waiting times at charging stations [[77], [78]]. Mixed integer linear programming (MILP) allows one to achieve mathematical optimization for problems including both continuous and discrete variables. Applied in EV optimization MILP maximizes charging schedules under consideration for grid constraints including capacity limits and ToU pricing. Deep reinforcement learning, or DRL, is the development of intelligent systems by means of deep learning coupled with reinforcement learning that learn optimal policies by means of experimentation। DRL allows dynamic response to changes in grid condition, user demand, and renewable energy availability adaptive charging strategies in electric vehicles. Appropriate for managing erratic elements in EV optimization, fuzzy logic is a decision-making tool handling uncertainty and ambiguity. For instance, fuzzy logic helps to regulate the variable output of renewable energy sources and maximizes charging strategies under uncertain conditions [[79], [80]].

The main objective functions engaged in EV optimization—maximizing grid stability, minimizing power losses, lowering waiting times at charging stations, and so lowering total charging costs—are examined in this part. Every objective function is explored together with the related limitations, so stressing the complexity and compromises involved in EV grid integration [81].

### 4.1 Maximize grid stability

The grid stability function aims to balance the demand for other loads including electric vehicles (EVs) with the power the grid supplies. Particularly when combining variable output renewable energy sources (such solar and wind), grid stability is quite important since changes in supply can cause imbalances [[82], [83]]. Maximizing grid stability ensures that the always sufficient available power meets EV charging needs without stressing the grid infrastructure or producing outages.

$$\text{Maximize}\;S=\sum_{i=1}^{N}\left(P_{\text{grid}}\left(t\right)-P_{\text{EVs}}\left(t\right)\right)$$

• Any EV charging system depends mostly on the capacity of the grid to deliver constant, reliable electricity. Particularly during peak charging times, EVs generate a great demand for the grid; hence, it is essential that the power supplied to the grid balances the power consumed by the vehicles. Any fluctuation can result in frequency changes that might induce brownouts or blackouts.
• Changing Power Flux: Where EV adoption is high, charging needs can cause grid instability via surges. Using vehicle-to----grid (V2G) systems will help to maximize stability by preventing these surges; also, scheduling and controlling EV charging behavior will enable the return of power to the grid as needed.

### 4.2 Constraints

#### 4.2.1 Power balance constraint

This constraint ensures that the grid continuously supplies at least as much energy as the EVs require. Balancing EV power consumption in real-time helps to avoid unbalance or shortages.

$$P_{\text{grid}}\left(t\right)\geq P_{\text{EVs}}\left(t\right)$$

#### 4.2.2 Maximum grid capacity

This limits guarantees stability or grid collapse free from possibility by making sure the power demand does not exceed the maximum capacity of the grid.

$$P_{\text{grid}}\left(t\right)\leq P_{\text{grid}}^{\max}$$

#### 4.2.3 Time-of-use (ToU) pricing

This constraint reflects time-of- use pricing, in which the cost of charging EVs changes with demand for electricity over the day. When grid demand is lower, off-peak charging is promoted, so preserving general grid stability.

$$C_{\text{charge}}\left(t\right)=C_{\text{base}}+C_{\text{peak}}\left(t\right)$$

### 4.3 Minimize power loss

Reducing power loss during transmission and distribution will help the EV charging system to run effectively. Usually, in transmission lines, the resistance results in power losses—also known as I2R losses. Reducing these losses will enable the maximum power that really reaches the EVs from the grid.

$$\text{Minimize}\;L=\sum_{i=1}^{N}\left(\frac{I^{2}R}{V_{\text{transmission}}}\right)$$

• As electricity flows across transmission and distribution lines, resistance in them causes some of the loss to be heat. Since losses are proportionate to the square of the current, it is especially concerning when many EVs are concurrently drawing power since it increases the current in the lines and hence causes the power losses.
• Furthermore influencing power losses in the transmission system is its voltage level. Higher voltage transmission systems typically suffer less losses over long distances, thus it is important to maintain suitable voltage levels across the grid especially as charging stations proliferate.

### 4.4 Constraints

#### 4.4.1 Current limitation

$$I\left(t\right)\leq I_{\max}$$

This constraint ensures that the current passing the transmission lines does not surpass the safe operating limit, so preventing overheating or maybe equipment breakdown.

#### 4.4.2 Voltage stability

$$V_{\text{transmission}}\left(t\right)\geq V_{\min}$$

This constraint ensures that the voltage never drops below a specified threshold, so avoiding inefficiencies in power supply and so affecting EV charging performance.

#### 4.4.3 Demand in power

$$P_{\text{delivered}}=P_{\text{grid}}-L$$

This ensures that the delivered electricity for the electric cars takes transmission losses into account. Keeping the losses low will help to maximize the output of the produced electricity.

### 4.5 *Minimize* waiting time

Minimal waiting times help to improve the user experience. This function seeks to reduce the time an electric vehicle has to wait before starting to charge at a charging station. Long waiting times compromise the convenience of owning an electric vehicle especially in metropolitan areas with lots of vehicles.

$$\text{Minimize}\;W=\sum_{i=1}^{N_{\text{EV}}}\left(t_{\text{arrival}}^{i}-t_{\text{start}\_\text{charge}}^{i}\right)$$

• User satisfaction directly affects the reduced waiting time at charging stations. EV owners expect a perfect charging experience, much as at regular gas stations. Adoption of electric vehicles can be greatly discouraged by delays caused by insufficient charging infrastructure or poor planning.
• By balancing demand and managing the volume of EVs that must be charged, optimization guarantees that charging stations are used effectively. Through better planning, the system can assist to reduce traffic at charging stations and improve the overall vehicle flow.

#### 4.5.1 Charging station capacity

This constraint ensures that the number of EVs charging at any one time does not exceed the physical capacity of the charging station, so preventing overload and reducing waiting times.

$$N_{\text{charging}}\left(t\right)\leq N_{\max}$$

#### 4.5.2 Minimum state of charge (SoC) to start charging

This constraint defines the level of charge (SoC) of an EV before beginning charge. It ensures that low SoC vehicles prioritize over those with sufficient charge, so reducing the likelihood of running out of any one vehicle.

$${\text{SoC}}^{i}\left(t\right)\leq {\text{SoC}}_{\min}$$

#### 4.5.3 Charging time

This constraint controls the charging time for an electric vehicle based on battery capacity and current so optimizing charging sessions to lower idle time at the station.

$$t_{\text{charge}}^{i}=\frac{{\text{Capacity}}_{\text{battery}}^{i}\times \left({\text{SoC}}_{\max}^{i}-{\text{SoC}}^{i}\left(t\right)\right)}{P_{\text{station}}}$$

### 4.6 Minimize Charging cost

Through implementing time-of- use (ToU) pricing, this function seeks to minimize the overall cost of charging all electric vehicles. ToU pricing varies the cost of electricity depending on demand by lower rates during off-peak hours and higher rates during periods of maximum demand.

$$\text{Minimize}\;C_{\text{total}}=\sum_{i=1}^{N_{\text{EV}}}\left(C_{\text{charge}}^{i}\times t_{\text{charge}}^{i}\right)$$

• When EVs are charged—that is, during off-peak hours—that lets the system take advantage of lower electricity rates, so saving costs for the grid operator as well as for consumers. This also drives a more in line demand curve for energy consumption.
• Long-Term Fiscal Management: Using electric cars depends on lowering charging costs since running expenses mostly influence user decisions. Dividing demand over several times of the day helps to maximize costs, so supporting grid stability as well as making EV ownership more reasonably priced.

#### 4.6.1 ToU price limits

By ensuring that EVs are not charged when electricity prices are at their highest, this constraint guarantees that total expenses are low even during peak hours.

$$C_{\text{charge}}^{i}\left(t\right)\leq C_{\max}\;\text{if}\;t\in \text{peak}\;\text{hours}$$

#### 4.6.2 Charging power constraint

This constraint ensures that the consumed power by the electric vehicle does not exceed the maximum capacity of the charging station, so preventing inefficiencies and too high costs.

$$P_{\text{EV}}\left(t\right)\leq P_{\text{station}}\left(t\right)$$

### 4.7 Combined multi-objective optimization

A multi-objective optimization technique can be used to balance trade-offs considering the several goals (grid stability, power loss, waiting time, and cost). Usually, this is stated as the weighted sum of the several goals:

$$\text{Minimize}\;F=w_{1}\cdot \frac{1}{S}+w_{2}\cdot L+w_{3}\cdot W+w_{4}\cdot C_{\text{total}}$$

$w_{1},w_{2},w_{3},w_{4}$ are weights representing each objective functions.

The state of charge (SoC) of each EV should be within limits:

$${\text{SoC}}_{\min}\leq \text{SoC}\left(t\right)\leq {\text{SoC}}_{\max}$$

The total energy supplied by the grid must equal the total energy consumed by EVs and the losses in the system:

$$\sum_{t=1}^{T}P_{\text{grid}}\left(t\right)=\sum_{t=1}^{T}P_{\text{EVs}}\left(t\right)+L$$

Optimizing electric vehicle charging offers a multi-dimensional challenge needing careful evaluation of many elements including grid stability, power loss, waiting time, and charging cost. From a balance between these opposing objectives attained by using a mix of sophisticated optimization techniques and well defined constraints, more effective EV operations and more user satisfaction follow. The given objective functions and constraints provide a good structure for addressing the primary challenges of EV integration into modern power grids. As the acceptance of EVs keeps rising, further developments in optimization techniques will be absolutely vital to ensure that the transportation and energy sectors can coexist peacefully and so support world sustainability goals.

### 4.8 Summary of various optimization methods

A recent review of optimization techniques used to electric vehicle (EV) charging systems and related energy management strategies evaluated several algorithms and approaches depending on their advantages, constraints, and specific use cases. Generally used in building-integrated microgrid (MG) systems, Finite Horizon Scheduling [84] has shown to ensure 85 % of energy needs are met by photovoltaic (PV) systems and can handle uncertainties, even if it needs improvement in forecasting accuracy and does not support bidirectional energy flow. Stochastic Integer Programming (SIP) [85] improves system security by letting a balance between robustness and economy; yet, it suffers with handling stochastic parameters including power availability and prices, especially in the framework of electric vehicle power grid systems. Although it might not be suitable for long-term operational strategies, especially in EV charging strategy optimizations, the One-Slot Look Ahead (OSLA) method [86] helps reduce running costs and increase local generating usage.

Though it provides complexity in implementation and parameter selection, mostly in EV microgrid management systems, the Self- Adaptive Modified Clonal Selection Algorithm (SAMCSA) [87] lowers general operational costs and improves mobile storage use. Usually used in residential building energy systems, Particle Swarm Optimization (PSO) [88] depends on further integration of market information and economic analysis but maintains user comfort with low energy consumption. Mixed Integer Linear Programming (MILP) [89] is efficient in reducing electricity costs and managing the State of Charge (SoC) of EVs even if demand response programs and smart homes should also be included.

Moreover, DETECt 2.3 [90] fits for policy development and achieves energy savings between 45 and 77 %; nevertheless, more solid energy policies are needed to support more general use. Although it lacks real EV use data and does not integrate renewable energy sources (RES), Model Predictive Control (MPC) combined with Optimal Control with Minimum Cost and Flexibility (OCCF) [91] maximizes the flexibility of EV charging stations and reduces operational costs, so limiting their effectiveness in EV charging station management even if it lacks real EV use data and does not integrate renewable energy sources (RES). Though more research on its use to EV inclusion and microgrid management is needed, the Epsilon Constraint method with a Fuzzy Satisfying Approach [92] efficiently manages uncertainty and has possibility for islanded operation.

Affected by battery degradation and feed-in rates, especially in V2G systems, the Genetic Algorithm (GA) [93] shows the possible advantages of Vehicle-to- Grid (V2G) technology and cost reductions. Although user parameter setting may restrict flexibility, linear programming [94] maximizes self-sufficiency and optimizes simultaneous EV charging, so more relevant to self-sufficient energy systems. Although more study is needed to evaluate its economic viability, particularly in residential building power contracting, the Mixed Binary Linear Problem (MBLP) combined with GA [95] optimizes power contracting and reduces electrical consumption by 47 %.

While MILP [96] maximizes RES use and minimizes imported energy, MILP for office building energy management [97] efficiently injects power into the grid; expansion to larger systems is necessary even if it requires performance comparisons with other approaches. Distributed Approximate Dynamic Programming (D-ADP) [98] achieves economic gains without sacrificing comfort levels, although it requires a sophisticated configuration and accurate data. More research including price elasticity and user behavior is required even if MILP [99] helps to reduce peak system load and EV charging costs.

NSGA-II [100] reduces greenhouse gas (GHG) emissions and achieves energy savings even if its high computational demand is a drawback especially when tackling several objectives. Mixed Integer Nonlinear Programming (MINLP) [101] lowers energy costs and GHG emissions based on Time-of- Use (ToU) tariffs, even if it does not solve ambiguity in EV driving behavior and electricity pricing. Although it requires more validation in bigger, more complex scenarios—especially in hybrid energy storage systems—the Artificial Bee Colony (ABC) [102] improves system efficiency and security while reducing total costs. Last but not least, although real-world testing is essential, the Gradient Tree Boosting Ensemble (GTBE) [103] reduces electricity costs and achieves a high generating-to- demand ratio; hence, it is suitable for building energy consumption optimization even if it might overfit in complex datasets. Considering the range of approaches needed to satisfy the several needs of EV integration, energy management, and grid stability, each of these technologies shows unique strengths and challenges.

## 5 Future scope of research

There are still several important directions for future research and development in EV technologies, battery systems, charging methods, and charge scheduling optimization even if acceptance of electric vehicles (EVs) keeps accelerating and technological developments unfold. By closing these gaps, one will be able to fully realize the possibilities of electric vehicles and overcome present obstacles on their integration into contemporary transportation and energy systems.

### 5.1 Advancements in battery technologies

With the major developments in battery technologies, next studies should focus on increasing energy density, charging speed, and lifetime [104]. The creation of solid-state batteries—which offer faster charging times and higher energy densities than lithium-ion batteries—piques especially great interest. Research on alternative battery chemistries, including lithium-sulfur batteries and metal-air batteries, might also offer better performance at less costs. Moreover under continuous focus should be initiatives on raising battery recyclability and developing second-life applications so that former EV batteries could be applied for energy storage in various fields.

Battery management systems (BMS) will especially need more development especially in connection with combining artificial intelligence and machine learning to enhance predictive maintenance and the real-time optimization of battery use. Moreover, as battery technology advances new safety protocols and legal frameworks will become increasingly important to ensure the safe application of next-generation batteries in mass-market electric cars [[105], [106]].

### 5.2 Smart charging infrastructure and wireless charging

Research on smart charging infrastructure will help to support the wide deployment of electric vehicles. Future charging stations have to be able to manage higher charging rates, provide better dependability, and interface easily with renewable energy sources. Development of smart charging technologies should help Vehicle-to- Grid (V2G) systems to more effectively support EVs as distributed storage units for the grid, so supporting grid balancing and stability. Furthermore, bi-directional charging technology demands more study particularly on its financial viability and impact on battery condition.

Wireless charging is still another promising area of research for next years. By eliminating the need for frequent stops at charging stations, dynamic wireless charging—where EVs could charge while in motion—may greatly enhance user experience. Research should focus on improving the efficiency, safety, and scalability of wireless charging systems as well as on conquering challenges with vehicle alignment, electromagnetic interference, and infrastructure costs.

### 5.3 Optimization of charging schedules

As EV acceptance rises, charge scheduling techniques have to become more dynamic and flexible. Real-time optimization algorithms that can adjust to fast changing conditions—such as shifting grid loads, varying electricity prices, and user preferences top priority—should then be the focus of next research. These optimization techniques will enable systems to forecast user behavior and modify charging schedules by means of artificial intelligence (AI) and machine learning (ML), so impacting grid dependability and hence reducing costs. Research should also look at multi-objective optimization techniques able to balance competing goals including lowering environmental impact, minimizing charging time, and optimizing grid stability. Moreover, including renewable energy sources into these optimization strategies will enable EVs charge from green energy when it is most available.

### 5.4 Integration with autonomous vehicles and mobility-as-a-service (MaaS)

The combination of electric vehicles (EVs) with autonomous vehicles (AVs) and Mobility-as- a- Service (MaaS) creates new research prospects. Autonomous electric vehicles could be tuned to charge sites either off-peak or with additional renewable energy. Future studies should look at ways to maximize efficiency, ease traffic at charging stations, and guarantee grid stability by changing AV fleets. Moreover, including EVs into MaaS systems might significantly decrease the number of privately owned vehicles, so reducing urban traffic and emissions. Research assures that MaaS vehicles run effectively and satisfy consumers; thus, optimal routing and charging of them depends on this. By real-time management of large fleets, this would demand advanced artificial intelligence-driven algorithms balancing vehicle demand, energy consumption, and environmental impact.

### 5.5 Cybersecurity and data privacy

Cybersecurity and user data protection will become absolutely vital as EVs get more linked and dependent on digital infrastructure. Future studies should focus on robust encryption techniques and safe communication systems protecting charging stations and electric cars from cyberattacks. Furthermore, safe data management systems have to be created to protect user privacy while yet enabling effective data sharing among platforms since cloud-based systems grow increasingly important for grid management and charge scheduling.

### 5.6 Life cycle assessment

Future studies in still another important field is the lifetime environmental impact of electric cars. Though EVs are touted as a sustainable replacement for conventional vehicles, the manufacturing, running, and disposal of EV components especially batteries, have major negative effects on the surroundings. Next research should give great weight to thorough life cycle studies assessing the carbon footprint, resource use, and waste management related with electric vehicles. Moreover, policies meant to increase the sustainability of EV manufacturing and recycling methods—particularly for batteries, should first focus on lowering the whole environmental impact of acceptance of EVs.

## 6 Conclusion

The developments in electric vehicle (EV) technologies, charging techniques, and optimization strategies indispensable for sustainable development have been investigated in this review. Growing adoption of electric vehicles calls for creative answers for problems with battery technology, grid integration, and charging infrastructure. From slow to wireless charging, several charging techniques are developing to satisfy growing needs; but, more work is required to improve efficiency and lower charging times. Driven by sophisticated algorithms, optimization techniques are essential for controlling costs, lowering power losses, and grid stabilization. Advancement of battery technologies, building smart charging infrastructure, and enhancement of real-time optimization algorithms should be the main priorities of next research. Future success of EVs depends also on integration with autonomous cars and Mobility-as- a- Service, together addressing cybersecurity and sustainability. In general, even if development has been achieved, more study and creativity are needed to overcome current obstacles and guarantee the sustainable expansion of electric cars in the worldwide transportation system.

## CRediT authorship contribution statement

**Vikram Goud Madaram:** Writing – original draft, Methodology, Formal analysis, Data curation, Conceptualization. **Pabitra Kumar Biswas:** Writing – review & editing, Supervision, Software, Project administration, Methodology, Investigation, Formal analysis. **Chiranjit Sain:** Validation, Supervision, Resources, Project administration, Methodology, Investigation. **Sudhakar Babu Thanikanti:** Writing – review & editing, Visualization, Validation, Software, Resources, Project administration, Methodology. **Praveen Kumar Balachandran:** Writing – review & editing, Validation, Supervision, Methodology, Investigation, Funding acquisition.

## Data availability statement

Not applicable.

## Funding

Authors have received no external funding.

## Declaration of competing interest

The authors declare the following financial interests/personal relationships which may be considered as potential competing interests:PKB (corresponding author) declares that he is currently holding a position on our editorial board. If there are other authors, they declare that they have no known competing financial interests or personal relationships that could have appeared to influence the work reported in this paper.
